# Targeting cryptic splicing variants in *SDCCAG8*, *CEP290*, and *ABCA4*: A translational workflow from diagnosis to ASO-based therapy

**DOI:** 10.1016/j.omtn.2026.103139

**Published:** 2026-10-14

**Authors:** Mariia Zadorozhna, Simona Rossi, Erika Tenderini, Ilaria Brentari, Anna Basile, Giulia Pezzin, Rebecca Venturini, Federica Isidori, Elena Luppi, Emanuele Panza, Fabio Sirchia, Elena Rossi, Mariateresa Zanobio, Marianthi Karali, Tommaso Pippucci, Giulio Piluso, Michela Alessandra Denti, Marco Seri, Sandro Banfi, Ivana Trapani, Elisa Giorgio

**Affiliations:** 1 Department of Molecular Medicine, University of Pavia, 27100 Pavia, Italy; 2 IRCCS Mondino Foundation, Neurogenetics Research Centre, 27100 Pavia, Italy; 3 Telethon Institute of Genetics and Medicine (TIGEM), Pozzuoli, Italy; 4 Scuola Superiore Meridionale (School of Advanced Studies), Genomics and Experimental Medicine Program (GEM), Naples, Italy; 5 Department of Cellular, Computational and Integrative Biology (CIBIO), University of Trento, 38123 Trento, Italy; 6 IRCCS Azienda Ospedaliero-Universitaria di Bologna, Bologna, Italy; 7 Department of Medical and Surgical Sciences, University of Bologna, Bologna, Italy; 8 UOSD Medical Genetics and Cardiomyology, Azienda Ospedaliera Universitaria Luigi Vanvitelli, Naples, Italy; 9 Medical Genetics, Department of Precision Medicine, University of Campania Luigi Vanvitelli, Naples, Italy; 10 Eye Clinic, Multidisciplinary Department of Medical, Surgical and Dental Sciences, University of Campania “Luigi Vanvitelli”, Naples, Italy; 11 Department of Advanced Biomedical Sciences, Federico II University, Naples, Italy; 12 Medical Genetics Unit, IRCCS San Matteo Foundation, Pavia, Italy

**Keywords:** MT: Oligonucleotides: Therapies and Applications, cryptic variant, antisense oligonucleotide, precision medicine, splicing modulation, inherited retinal diseases, retinal organoids, eye disorders

## Abstract

A definitive molecular diagnosis is essential for the clinical management of rare genetic diseases (RGDs), guiding prognosis, genetic counseling, and therapeutic development. RNA-based therapeutics have emerged as promising strategies for RGDs owing to their ability to modulate gene expression and splicing. Here, we establish an integrated and scalable pipeline from variant discovery to RNA-based therapy in inherited retinal diseases (IRDs). Genome sequencing identified three cryptic variants in known IRD genes. Two novel deep intronic variants in *SDCCAG8* were characterized by RT-PCR to induce pseudoexon inclusion. In addition, a previously described deep intronic variant in *CEP290* was detected and reproduced on patient-derived fibroblasts, confirming aberrant splicing. A third family carrying the recurrent *ABCA4* c.768G>T silent variant was included to corroborate the translational strength of our pipeline and to provide an additional model for RNA-based therapeutic correction. We designed and tested 16 splice-switching antisense oligonucleotides (ssASOs) in patient-derived fibroblasts, HeLa cells transfected with mutant minigenes, and, when available, human induced pluripotent stem cell (hiPSC)-derived retinal organoids. For each variant, at least one ssASO successfully improved normal splicing and increased gene expression in patient-derived models. Overall, this study validates an innovative end-to-end translational workflow and highlights how molecular diagnosis can directly drive the development of personalized RNA-based therapies.

## Graphical abstract

**Figure:**
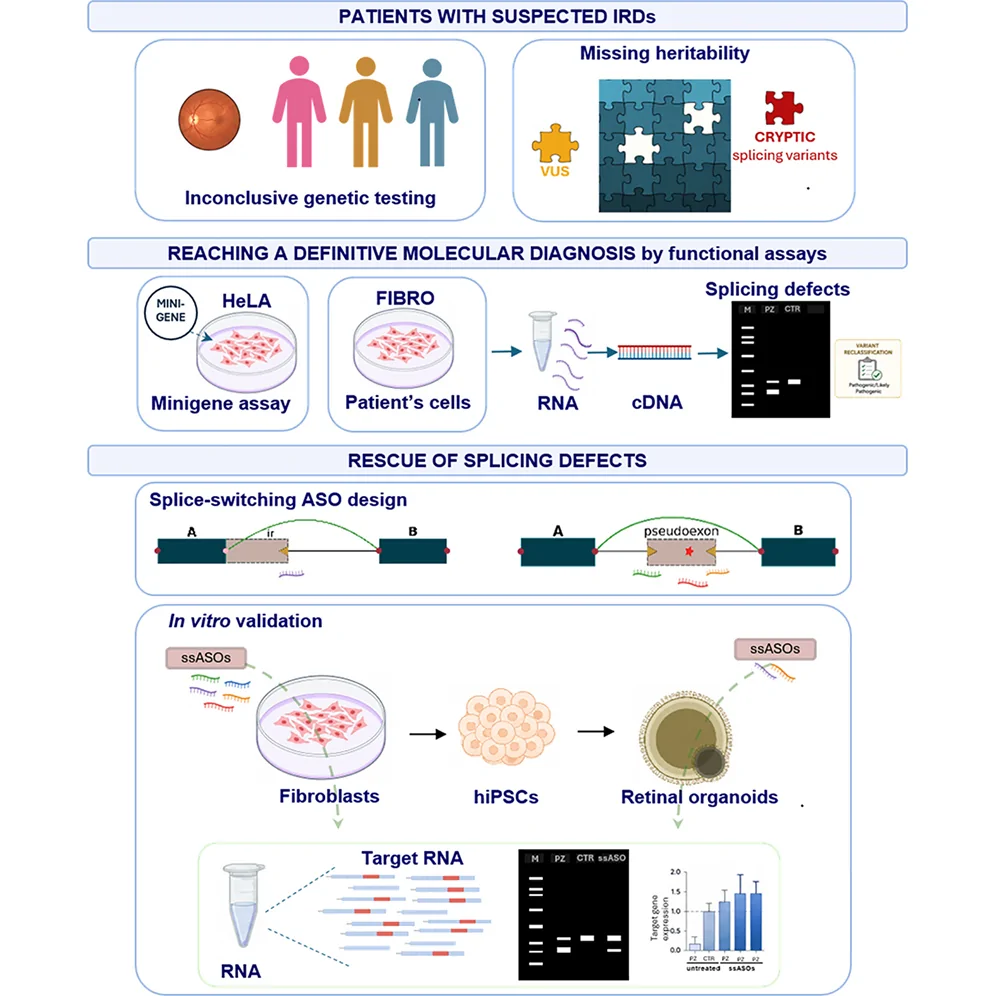

## Teaser

Zadorozhna and colleagues investigated three unsolved families affected by inherited retinal diseases (IRDs), identifying causal cryptic variants and designing ssASO therapeutic strategies. This study highlights the clinical utility of integrating genomic diagnostics with functional assays and supports the development of personalized RNA-based therapies for patients with currently untreatable IRDs.

## Introduction

Reaching a genetic diagnosis provides critical benefits, including accurate prognosis, informed genetic counseling, and the possibility of targeted therapeutic interventions. Although next-generation sequencing (NGS), particularly exome sequencing (ES), has substantially improved diagnostic rates for rare genetic diseases (RGDs), approximately 50% of patients still remain without a definitive molecular diagnosis.^1^ Several factors contribute to this diagnostic gap, but a significant proportion of the missing heritability is increasingly attributed to cryptic variants that alter RNA splicing or disrupt non-coding regulatory elements. These variants are often missed by ES but can be captured through genome sequencing (GS).^2^^,^^3^^,^^4^

Parallel to this challenge, only ∼5% of patients with RGDs currently have access to approved therapies,^5^ reflecting the limited and often individualized market for ultra-rare conditions. This unmet need has fueled growing interest in RNA-based therapeutics,^6^^,^^7^ a versatile and rapidly evolving drug class that gained widespread attention after the deployment of mRNA-based vaccines during the COVID-19 pandemic. RNA therapeutics encompass three major functional categories: (1) splice-switching compounds, such as splice-switching antisense oligonucleotides (ssASOs), which modulate exon inclusion or exclusion to correct aberrant splicing; (2) RNA-silencing molecules, which reduce the expression of disease-causing transcripts; and (3) RNA-upregulating strategies, which enhance transcription or translation to increase functional protein levels.^8^

To date, 19 oligonucleotide drugs have been approved by the FDA, including five ssASOs. Four, namely eteplirsen, golodirsen, viltolarsen, and casimersen, target splicing defects in Duchenne muscular dystrophy (DMD), while nusinersen is approved for spinal muscular atrophy (SMA).^8^ Beyond these, at least 28 patients have been treated in *n*-of-1 or *n*-of-few clinical settings with ASOs tailored to their unique pathogenic variants,^9^ including milasen for CLN7 Batten disease,^10^ atipeksen for ataxia-telangiectasia (A-T),^11^ jacifusen for amyotrophic lateral sclerosis (ALS),^12^ and valeriasen for developmental and epileptic encephalopathy type 14.^13^ These pioneering cases demonstrate the clinical feasibility of individualized RNA therapeutics but also highlight the crucial need for workflows that can rapidly identify cryptic splicing variants and evaluate their responsiveness to ssASO correction.

One of the major limitations of RNA-based therapeutics remains efficient drug delivery. While systemic administration is the most common route for RNA therapeutics in clinical practice, it is generally associated with limited delivery efficiency, with a large proportion of the administered dose accumulating in the liver and kidneys rather than reaching the target tissue.^7^ In contrast, RNA therapeutics for inherited retinal diseases (IRDs) benefit from local ocular administration, which minimizes systemic exposure while achieving high drug concentrations at the target site.^6^^,^^7^ However, not all retinal tissues are equally accessible to oligonucleotide-based therapies. ASO distribution depends on multiple factors, including the route of administration, oligonucleotide chemistry, and the anatomical barriers of the eye. Several delivery routes have been explored for targeting the posterior segment, including topical, systemic, periocular (e.g., subconjunctival and posterior juxtascleral), intravitreal, suprachoroidal, and subretinal administration.^14^ Topical and systemic administration provide limited drug exposure to the posterior segment because of the corneal and blood-retinal barriers, respectively, whereas periocular administration generally results in lower retinal bioavailability.^14^ Intravitreal injection is currently the most widely used route for delivering RNA therapeutics to the posterior segment because it bypasses these barriers, enables broad retinal distribution, and has demonstrated a favorable safety profile in both preclinical and clinical studies.

Although subretinal administration provides direct access to the photoreceptor layer and retinal pigment epithelium and may therefore enhance local drug exposure to these target cells, its greater surgical invasiveness and limited suitability for repeated dosing currently make intravitreal injection the more clinically practical route for ASO-based therapies.^14^ Notably, intravitreal administration has already demonstrated that ASOs can effectively reach photoreceptors and produce therapeutic benefit in IRDs, as exemplified by sepofarsen for CEP290-associated Leber congenital amaurosis (LCA).^15^^,^^16^

Senior-Løken syndrome (SLSN) is a rare autosomal-recessive ciliopathy (estimated incidence 1:1,000,000) characterized by retinal degeneration and nephronophthisis, ultimately leading to blindness and end-stage renal disease. Ten genes have been implicated, including *SDCCAG8*, which causes SLSN type 7 (SLSN7; MIM: 613615) and encodes an 88-kDa protein containing a nuclear localization signal and eight putative coiled-coil domains.^17^ SDCCAG8 localizes to the connecting cilium of photoreceptors, particularly the basal body and transition zone, where it contributes to ciliary function.^18^

LCA, a severe early-onset retinal degeneration affecting 2–3 per 100,000 newborns, is characterized by visual impairment from birth or early infancy, nystagmus, poor pupillary responses, the oculodigital sign, and severely abnormal or absent electroretinograms.^19^ Bi-allelic pathogenic variants in *CEP290* cause LCA type 10 (LCA10; MIM: 611755), the most common LCA subtype (about 30% of cases). *CEP290* encodes a key transition-zone protein that regulates ciliary protein trafficking and maintains the molecular barrier between the inner and outer segments of photoreceptors.^20^^,^^21^

Stargardt disease type 1 (STGD1; MIM: 248200) is yet another IRD, with a prevalence of approximately 1:10,000, caused by bi-allelic pathogenic variants in *ABCA4*.^22^ ABCA4 is an ATP-binding cassette transporter highly expressed in photoreceptors and essential for clearing toxic retinal by-products such as N-retinylidene-N-retinylethanolamine.^22^ Loss of ABCA4 function leads to accumulation of toxic bisretinoids in photoreceptors and the retinal pigment epithelium, driving progressive retinal degeneration.^23^

In this study, we aimed to evaluate the feasibility of an RNA-based therapeutic strategy for three patients with clinical suspicion of SLSN or LCA and a family with a molecular diagnosis of STGD1. Through GS, reverse transcription polymerase chain reaction (RT-PCR), minigene assays, and *in vitro* ssASO testing, we established a proof-of-concept workflow, spanning from variant discovery to therapeutic design, demonstrating the clinical utility and translational potential of an end-to-end pipeline for the identification and correction of splicing defects in IRDs.

## Results

### GS and *in silico* splicing prediction tools suggest a molecular diagnosis in the patients

Within the precision diagnosis and therapy for rare diseases by interpreting non-coding genomes (PARADIGM) project, GS identified cryptic variants in two IRD families (Table 1). The pathogenic effect of *CEP290*:c.6136−436A>G on splicing has been previously assessed.^25^ Concerning the two novel cryptic variants in *SDCCAG8*, *in silico* prediction tools suggested a causative role for only one of them through splicing alterations (Table 2). Specifically, Human Splicing Finder (HSF) analysis indicated that the *SDCCAG8*:c.740+288G>C variant induces a significant shift in the ratio of intronic splicing enhancers (ISEs) to intronic splicing silencers (ISSs), resulting in a predominance of enhancer motifs (Table 2). Consistently, SpliceAI predicted the activation of cryptic acceptor splice sites 53 nt upstream of the c.740+288G>C variant and a cryptic donor splice site 58 nt downstream (Table 2). In contrast, neither HSF nor SpliceAI predicted any splicing impact on splicing for the *SDCCAG8*:c.740 + 267C>T variant (Table 2). Additionally, a third IRD family (Table 1), previously described^26^^,^^27^ and carrying the recurrent *ABCA4* silent variant c.768G>T (Table 2), was included in the study to further support the feasibility of the diagnostic-to-therapeutic RNA-based pipeline.

**Table 1:** IRD families included in the study

Family	Samples ID	Status	Gene	Inheritance	Genotype	Previously reported (ClinVar ID/reference)
SLSN	SL1	proband	*SDCCAG8*	AR	c.740 + 267C>T c.740 + 288G>C	– –
SLSN	SL2	affected brother	*SDCCAG8*	AR	c.740 + 267C>T c.740 + 288G>C	– –
SLSN	SL3	mother	*SDCCAG8*	AR	c.740 + 267C>T WT	– –
SLSN	SL4	father	*SDCCAG8*	AR	c.740 + 288G>C WT	– –
LCA	LC1	proband	*CEP290*	AR	c.6604del c.6136 − 436A>G	197597^24^ Zeuli et al.^25^
LCA	LC2	mother	*CEP290*	AR	c.6604del WT	197597^24^ /
LCA	LC3	father	*CEP290*	AR	c.6136 − 436A>G WT	Zeuli et al.^25^ /
STGD1	ST1	proband	*ABCA4*	AR	c.768G>T c.768G>T	99505^26^^,^^27^ 99505^26^^,^^27^

For variants previously described in the literature, the corresponding ClinVar ID and reference are reported. –, novel variants; /, WT allele; AR, autosomal recessive; WT, wild type.

**Table 2:** *In silico* predictions of cryptic variants included in the study

	Intron	HSF	SpliceAI	*In vitro* validation
***SDCCAG8***				

c.740+267C>T	7	no effect predicted	no effect predicted	causative: pseudoexon inclusion (ψ7a; 112 bp)
c.740+288G>C	7	ISE/ISS motifs ratio alteration	acceptor gain (−53 bp); donor gain (58 bp)	causative: pseudoexon inclusion (ψ7a; 112 bp)

***CEP290***				

c.6136−436A>G	44	ISE/ISS motifs ratio alteration	acceptor gain (−62 bp); donor gain (34 bp)	causative: pseudoexon inclusion (ψ44; 97 bp)

***ABCA4***				

c.768G>T	6	broken WT donor site	donor loss (0 bp); donor gain (35 bp)	causative: intron retention (ir6; 35 bp)

Predicted effects of the cryptic variants are shown based on Human Splicing Finder (HSF; https://hsf.genomnis.com/home) and SpliceAI (https://www.introni.it/splicing.html), along with results from *in vitro* functional validation. See Figure 1 for more details.

### *In vitro* experiments confirm the pathogenicity of *SDCCAG8* cryptic variants through splicing anomalies

Amplification and electrophoretic analysis of the target *SDCCAG8* cDNA region showed two main amplicons in the control sample, namely a prominent lower band and a faint intermediate band, and three amplicons in the patient sample (Figure 1A). Indeed, the patient displayed both an additional upper band and reduced intensity of the lower band compared to the control (Figure 1A). TA cloning and Sanger sequencing revealed two physiological splicing isoforms shared by controls and the patient samples (7-8 and 7-7b-8), and two patient-specific isoforms generated by inclusion of a 112 bp pseudoexon (ψ7a) (7-ψ7a-8 and 7-ψ7a-7b-8) (Figure 1A). Pseudoexon ψ7a introduces an out-of-frame insertion leading to a premature stop codon after 42 amino acids [p.(Arg247ArgfsTer42)]. Of note, ψ7a contains both cryptic variants, enabling confirmation of their causative role. Indeed, we identified colonies carrying the wild-type (WT) and the mutated allele at c.740 + 267 and c.740 + 288, respectively, and vice versa (Figure 1A). SLSN-specific splicing anomalies identified in a patient’s fibroblasts represent a primary readout for evaluating ASO treatments.

**Figure 1:**
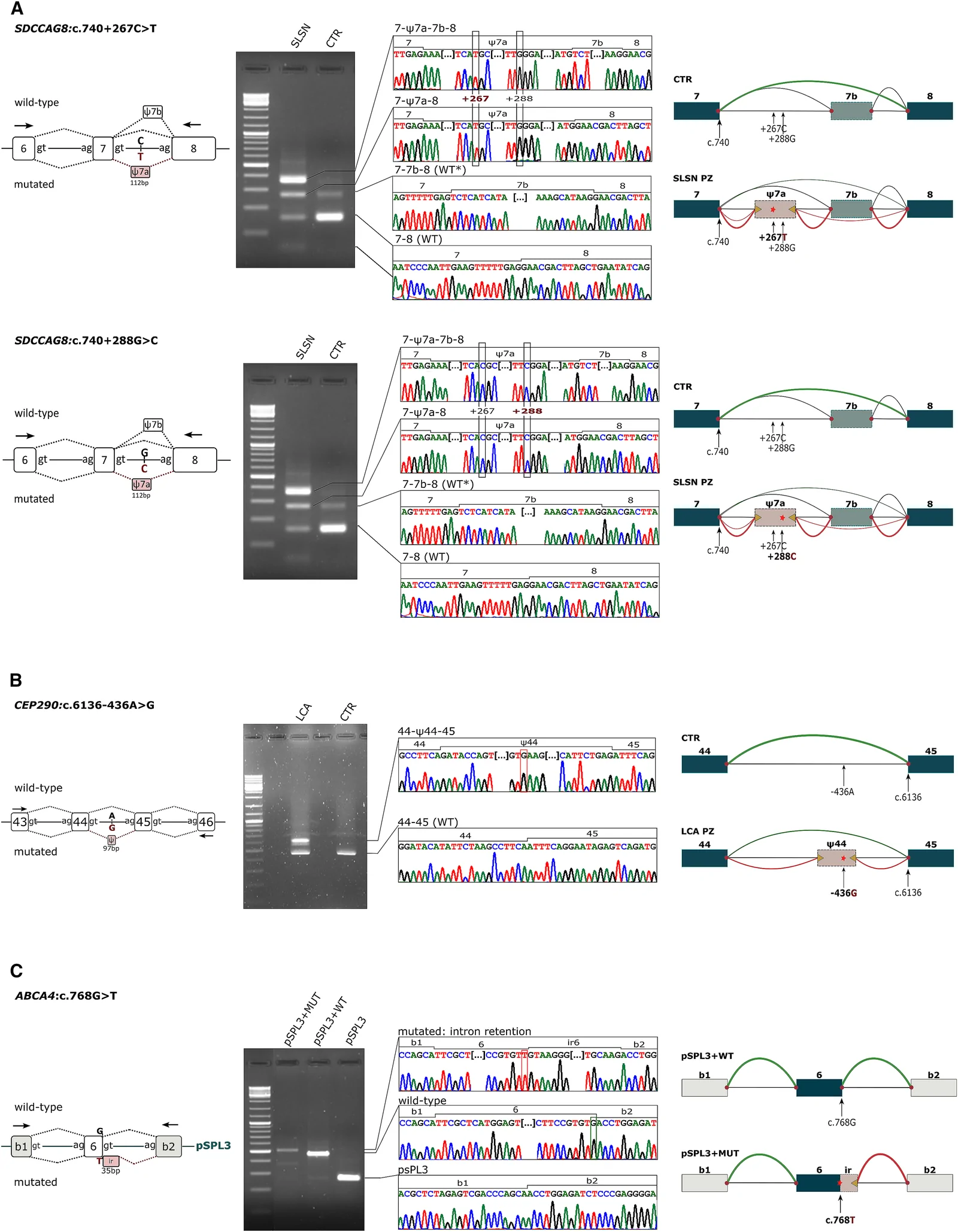
Characterization of splicing anomalies associated with cryptic variants in *SDCCAG8*, *CEP290*, and *ABCA4* genes Each image shows a schematic representation of splicing isoforms (left), electrophoretic analysis of the amplified target cDNA region (middle), and electropherograms of the corresponding bands with schematic representations of the observed isoforms (right). PZ, patient; CTR, control. Arrows: primers exploited for RT-PCR experiments. (A) Direct RNA analysis of *SDCCAG8* variants c.740+267C>G (top) and c.740+288C>T (bottom) in the SLSN-SL1 case: two physiological isoforms (7-8 and 7-7b-8) were detected in both control and patient samples, along with two patient-specific isoforms (7-ψ7a-8 and 7-ψ7a-7b-8), generated by inclusion of a 112 bp pseudoexon (ψ7a). Both variants caused the inclusion of the same pseudoexon. The electrophoretic image is intentionally reused in the top and bottom images because both analyses originated from the same initial RT-PCR experiment. The RT-PCR products were subsequently subjected to TA cloning to separate transcripts derived from the maternal and the paternal alleles, enabling allele-specific characterization of the splicing isoforms associated with the c.740+267C>G (top) and c.740+288C>T (bottom) variants, respectively. (B) Direct RNA analysis of *CEP290* variant c.6136−436A>G in the LCA-LC1 case: a physiological isoform (44-45) and an additional patient-specific isoform (44-ψ44-45), generated by inclusion of a 97 bp pseudoexon (ψ44), were detected, reproducing the previously reported splicing defect and providing a cellular readout for ASO testing. (C) Minigene assay of *ABCA4* silent variant c.768G>T. The mutant construct introduces a cryptic donor splice site downstream of exon 6, resulting in 36 bp intron 6 retention (b1-6-ir6-b2), consistent with previous reports and enabling establishment of a splicing readout suitable for ASO-mediated correction.

### LCA fibroblasts and HeLa cells expressing the ABCA4 minigene provide cellular models suitable for ASO testing

Amplification and electrophoretic analysis of the target *CEP290* cDNA showed a single amplicon in the control and two in the patient, one of which was shared with the control (Figure 1B). Amplicon characterization by TA cloning and sequencing of individual white colonies identified the physiological isoform (44-45) and a patient-specific isoform including a 97 bp pseudoexon (ψ44) (44-ψ44-45) (Figure 1B), in accordance with previous reports.^25^ Pseudoexon ψ44 generates an out-of-frame insertion with a premature stop codon after 32 amino acids [p.(Ile2046AspfsTer32)] (Figure 1B).

For the *ABCA4* variant, HeLa cells expressing the WT minigene construct (pSPL3+ABCA4-WT) produced the expected physiological isoform (b1-6-b2), whereas the mutant construct (pSPL3+ABCA4-MUT) generated a slightly larger amplicon (Figure 1C). Sanger sequencing confirmed the retention of 35 nt from intron 6 (ir6), resulting in an out-of-frame insertion and a premature stop codon after 17 amino acids [p.(Leu257ValfsTer17)] (Figure 1C), as previously reported.^26^ The LCA- and STGD1-specific splicing anomalies reproduced in patient-derived fibroblasts or in HeLa cells expressing the minigene system provide robust readouts for evaluating ASO-mediated splicing correction.

### All cryptic variants are targetable by ssASO strategies

We considered four different strategies to rescue the *SDCCAG8* splicing anomaly, namely targeting the cryptic acceptor (1) or donor (2) splice sites of ψ7a or masking the two variants, namely c.740+267C>T (3) and the c.740+288G>C (4) (Figure 2A). These four strategies allowed the design of a total of 17 ASOs. Following RT-PCR results showing the abundance of the aberrant 7-ψ7a-7b-8 isoform in the patient, we decided to also target the donor splice site of exon 7b (5) (Figure 2A) by 4 ASOs (Table 4). Candidate prioritization criteria based on sequence similarity, GC content, and thermodynamic properties (secondary analyses) led to the exclusion of 17 ssASOs from further consideration (Table 4). As a result, four ASOs derived from strategies 1 and 5 were synthesized. For the *CEP290* variant, we considered three rescuing strategies, namely targeting the cryptic acceptor (1) or donor (2) splice sites of ψ44 or (3) masking the variant c.6136−436A>G (Figure 2B). A total of 11 ASOs were designed, and following secondary analyses, 7 molecules were selected for synthesis (Table 4). Finally, targeting the cryptic donor splice site was considered the paramount strategy for *ABCA4* splicing anomaly (Figure 2C), allowing the design of 9 ASOs, five of which were selected for synthesis (Table 4). Additionally, five different scramble (SCR) molecules of the same length (range: 22–26 nt) and identical chemical modifications to the therapeutic ASOs were designed (Table 4).

**Figure 2:**
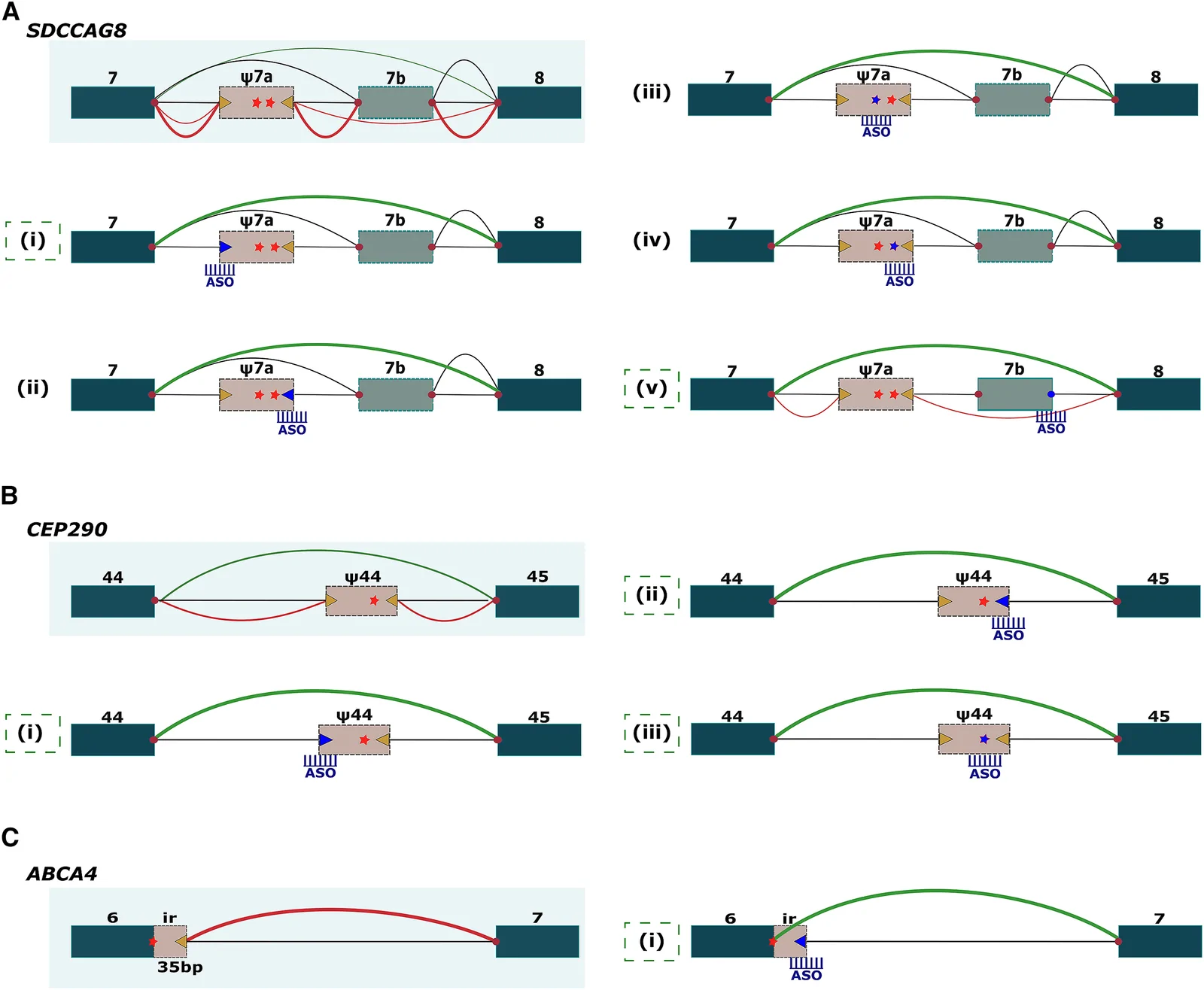
Overview of the splice-switching ASO strategies for rescuing aberrant splicing in *SDCCAG8*, *CEP290*, and *ABCA4* genes For each gene, a schematic representation of splicing isoforms identified in patient samples before treatment is shown (boxed with shaded light-blue rectangles). Red lines represent variant-specific splicing events, while green or black lines correspond to physiological splicing isoforms (also observed in control samples). Line thickness indicates the relative abundance of each splicing event. (A) For *SDCCAG8* variants, five ssASO-based strategies were designed: (i) blocking the cryptic acceptor splice site of ψ7a, (ii) blocking the cryptic donor splice site of ψ7a, (iii) masking the c.740+267C>T variant, (iv) masking the c.740+288G>C variant, and (v) targeting the donor splice site of exon 7b. (B) For the *CEP290* variant, three ssASO strategies were developed: (i) blocking the cryptic acceptor splice site of ψ44, (ii) blocking the cryptic donor splice site of ψ44, and (iii) masking the c.6136−436A>G variant. (C) For the *ABCA4* variant, a single strategy was designed to block the cryptic donor splice site. In all images, ssASO strategies selected for experimental validation are highlighted with green dashed rectangles.

### ssASOs effectively modulate splicing anomalies *in vitro*

We assessed ssASO (Table 4) efficacy after 48-h treatment of patient fibroblasts (SLSN-SL1 and LCA-LC1) or HeLa cells transfected with pSPL3+ABCA4-MUT (STGD1 case). In the SLSN family (SL1), ssASOs AC3 and AC4 significantly increased physiological *SDCCAG8* splicing relative to mock-treated or scramble controls while reducing aberrant isoforms. In contrast, ssASOs DO19 and DO20 produced only modest increases in physiological splicing and partially reduced the aberrant 7-ψ7a-7b-8 isoform, with persistent expression of 7-ψ7a-8. Scrambled ASOs had no detectable effect (Figure 3A). Semi-quantitative densitometry confirmed these findings (Figure 3B), showing significantly higher levels of the physiological isoform after AC3 and AC4 treatment compared to mock- or scramble-treated samples (percentage of physiological isoform: mean ± standard error of the mean [SEM]; control [CTR]: [mock 92 ± 3]; patient [PZ]: [mock 23 ± 5; 65 nM AC3 70 ± 4; AC4 71 ± 3]; Table S1) (scramble vs. AC3 *p* = 0.0079; scramble vs. AC4 *p* = 0.0079) (Figure 3B; Table S1).

**Figure 3:**
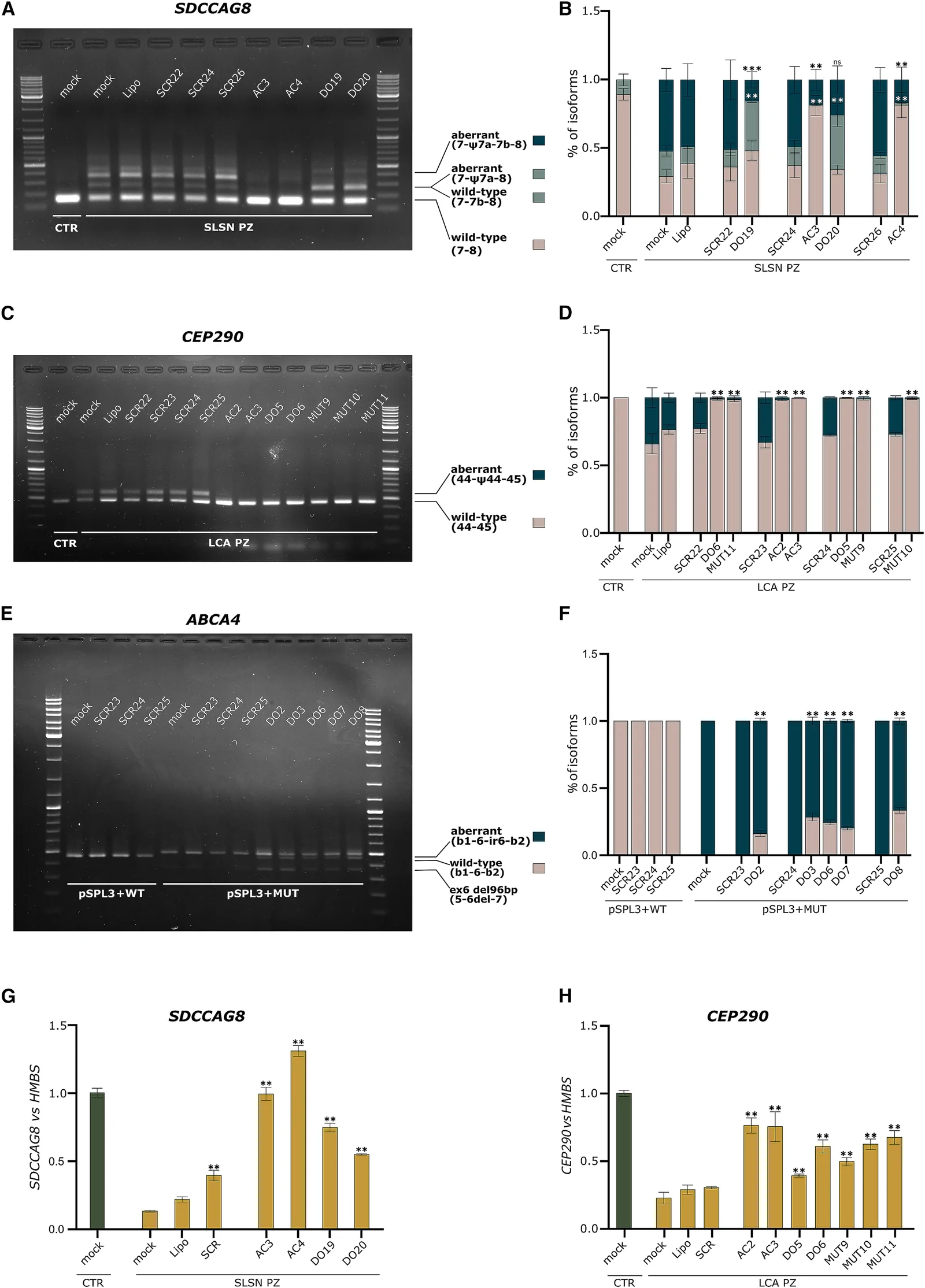
*In vitro* validation of ssASOs molecules Fibroblasts derived from patients (SLSN and LCA cases) or HeLa cells transfected with the pSPL3+ABCA4-MUT plasmid (STGD1 case) were untreated (mock) or treated with either vehicle (Lipo), scramble ASOs (SCR), or therapeutic ssASOs at final concentrations of 20, 35, 50, or 65 nM for 48 h. At least three independent technical replicates were performed. Electrophoretic analysis of RT-PCR products is shown for *SDCCAG8* (A), *CEP290* (C), and *ABCA4* (E) following 20 nM treatment, as exemplification (B, D, and F). Semi-quantitative densitometric analysis of splicing isoforms was performed. Statistical significance (indicated by asterisks) was determined by comparing ssASO-treated samples to length-matched scramble controls (∗∗*p* < 0.01 and ∗∗∗*p* < 0.001; two-tailed Mann-Whitney test). (G and H) Quantitative RT-PCR analysis of *SDCCAG8* and *CEP290* expression levels. Fold change and standard error of the mean (SEM) are reported. Statistical significance is shown comparing treated samples to mock (∗∗*p* < 0.01; two-tailed Mann-Whitney test). PZ, patient-derived fibroblasts; CTR, control-derived fibroblasts.

In the LCA family (LC1), all seven *CEP290*-targeting ssASOs markedly reduced the pathological isoform and promoted the physiological one at all concentrations tested (Figure 3C; Table S2). Densitometry confirmed rescue to near-control levels (Figure 3D), corroborating the therapeutic potential of all ssASOs (percentage of physiological isoform: mean ± SEM; CTR: [mock 100 ± 0]; PZ: [mock 66 ± 7; 65 nM AC2 98 ± 1; AC3 97 ± 2; DO5 99 ± 0; DO6 99 ± 0; MUT9 100 ± 0; MUT10 100 ± 0; MUT11 99 ± 0]; Table S2). Statistical analysis showed significant improvement for all *CEP290*-targeting ssASOs (*p* = 0.0079) (Figure 3D; Table S2).

Finally, the five *ABCA4*-targeting ssASOs partially restored physiological splicing in the ABCA4 minigene model (Figures 3E and 3F). Notably, a smaller transcript was detected, and its expression increased upon ASO treatment, consistent with previous observations.^27^ Densitometry and statistical analysis supported their activity (percentage of physiological isoform: mean ± SEM; WT: [mock 100 ± 0]; MUT: [mock 0 ± 0; 65 nM DO2 42 ± 12; DO3 36 ± 11; DO6 34 ± 11; DO7 39 ± 11; DO8 37 ± 12]) (scramble vs. ssASOs *p* = 0.0079) (Figure 3F; Table S3).

Quantitative RT-PCR confirmed that ssASO treatment significantly increased total *SDCCAG8* and *CEP290* mRNA levels at the lowest concentration tested (20 nM), supporting the therapeutic relevance (*SDCCAG8* vs. hydroxymethylbilane synthase [*HMBS*] expression: mean ± SEM; CTR: [mock 1.003 ± 0.034]; PZ: [mock 0.133 ± 0.007; SCR 0.220 ± 0.020; 20 nM AC3 0.995 ± 0.048; AC4 1.311 ± 0.040; D19 0.748 ± 0.032; DO20 0.550 ± 0.005]; *CEP290* vs. *HMBS* expression: mean ± SEM; CTR: [mock 1.001 ± 0.022]; PZ: [mock 0.228 ± 0.043; SCR 0.305 ± 0.008; 20 nM AC2 0.764 ± 0.057; AC3 0.755 ± 0.111; DO5 0.394 ± 0.012; DO6 0.609 ± 0.049; MUT9 0.497 ± 0.031; MUT10 0.626 ± 0.039; MUT11 0.676 ± 0.051]) (Figures 3G and 3H; Table S4).

### *ABCA4*-targeting ssASOs modulate splicing anomalies in patient-derived retinal organoids

To further corroborate the therapeutic potential of *ABCA4*-targeting ssASOs in a patient-derived system, STGD1 human induced pluripotent stem cell (hiPSC)-derived retinal organoids (ROs) (Figure 4A) were gymnotically treated for 4 weeks with a pool of the two most effective ssASOs (DO3 and DO8, ABCA4 ASOs). In parallel, ROs were treated with a recently reported ssASO developed by Karjosukarso et al.,^27^ which also targets the cryptic donor splice site of the intron retention fragment (ir6) (C+_1; positive control), or with scramble molecules. The *ABCA4* ASOs partially restored physiological splicing in STGD1 ROs (Figures 4B and 4C), showing an effect at least comparable to that observed with the positive control ASO (C+_1). As expected, the smaller transcript was detected specifically in patient-derived ROs and its expression increased upon ASO treatment.^27^ Sanger sequencing confirmed that this transcript corresponds to a deletion of the last 96 bp of *ABCA4* exon 6 (c.673_768del). Densitometric analysis and statistical evaluation supported the activity of *ABCA4* ASOs (percentage of physiological isoform: mean ± SEM; CTR: [SCR 100 ± 0]; STGD1: [SCR 0 ± 0; ABCA4 ASOs 14.2 ± 2.5; C+_1 11.7 ± 1.8]) (scramble vs. ssASOs *p* = 0.0061) (Figure 4C; Table S5). SYBR Green-based qPCR analysis further corroborated the ASO-mediated modulation of splicing in STGD1 ROs (percentage of physiological isoform: mean ± SEM; CTR: [SCR 96.3 ± 1.68]; STGD1: [SCR 8.00 ± 1.88; ABCA4 ASOs 18.2 ± 0.77; C+_1 20.6 ± 4.83]) (scramble vs. ssASOs *p* = 0.0286) (Figures 4D and 4E; Table S5). Based on published data,^27^ we synthesized the positive control ASO (C+) with two different chemical modifications, namely 2′-O-methyl RNA phosphorothioate (2′OMePS; C+_1) and 2′-O-methoxyethyl phosphorothioate (2′MOE/PS; C+_2). Our data indicate that the 2′MOE/PS modification is more effective in modulating splicing patterns in hiPSC-derived ROs (percentage of physiological isoform: mean ± SEM [C+_1 11.7 ± 1.8; C+_2 36.9 ± 3.8 ssASOs]) (C+_1 vs. C+_2 *p* = 0.0159) (Figure S2; Table S5).

**Figure 4:**
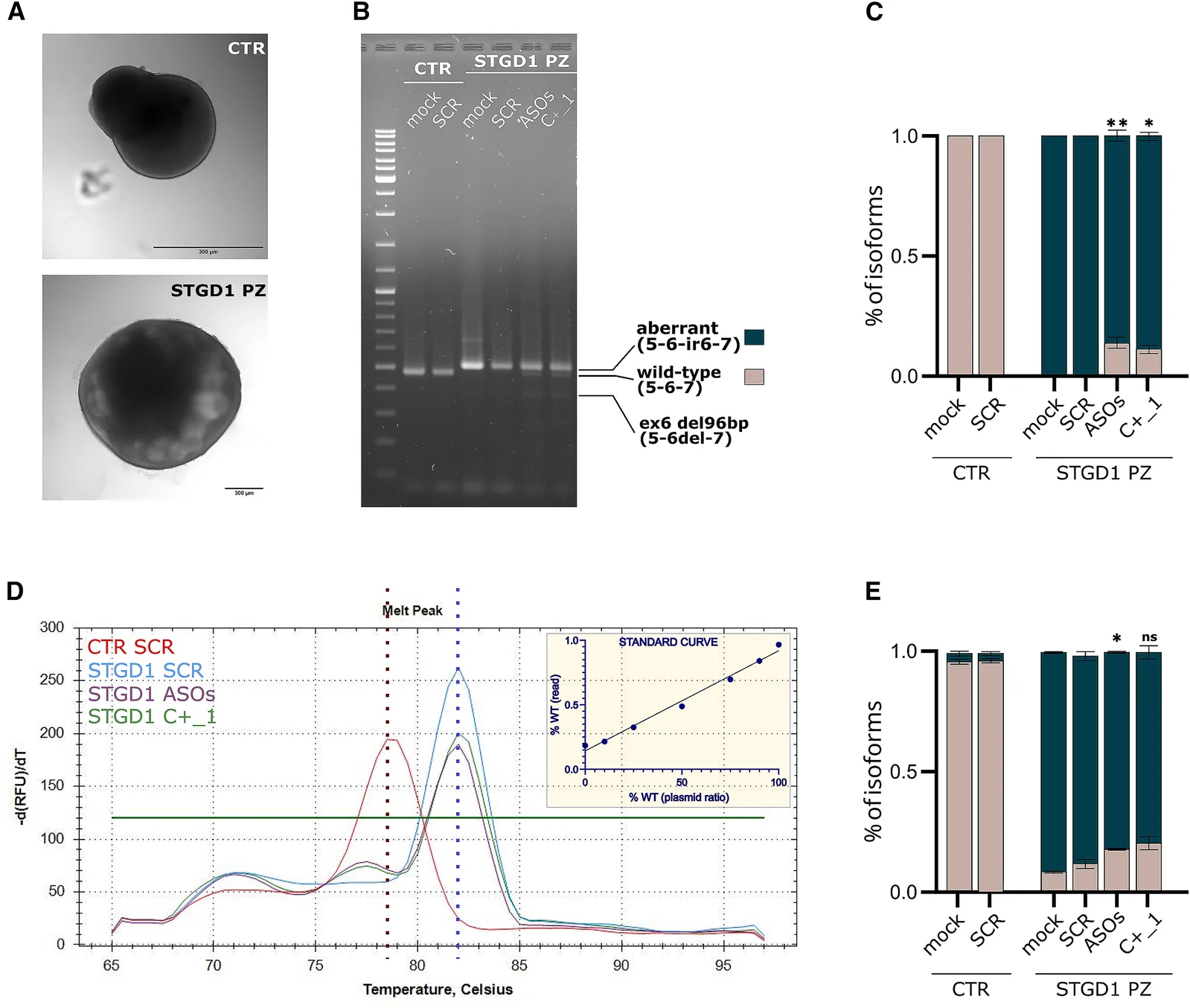
*In vitro* validation of *ABCA4* ssASOs in hiPSC-derived retinal organoids (A–D) 130-day-old retinal organoids (A) derived from a STGD1 patient were left untreated (mock) or gymnotically treated with scramble ASOs (SCR), a pool of therapeutic DO3 and DO8 ASOs (ASOs), and a positive control ASO (C+_1 from Karjosukarso et al.^27^) at final concentrations of 10 μM twice per week for 4 weeks. Electrophoretic analysis of RT-PCR products (B) and semi-quantitative densitometric analysis of splicing isoforms (C) are shown. Statistical significance (indicated by asterisks) was determined by comparing ASO-treated samples with scramble controls (∗*p* < 0.05 and ∗∗*p* < 0.01; two-tailed Mann-Whitney test). (D) Representative SYBR Green-based qPCR melting curves for control (CTR) and STGD1 samples are shown. Melting peaks for the physiological isoform (WT) correspond to Tm of 78.5°C (red dotted line), whereas those for the mutated isoform (MUT) correspond to a Tm of 82°C (light-blue dotted line). The standard curve generated from plasmid mixture analysis is shown (R^2^: 0.9884; see Figure S1 for details). (E) Normalized d(RFU)/dT values were interpolated against the standard curve to determine WT:MUT transcript ratios for each sample (percentage of isoform-interpolated values). Statistical significance (indicated by asterisks) was determined by comparing ssASO-treated samples with scramble controls (∗*p* < 0.05; two-tailed Mann-Whitney test). CTR, control retinal organoids; STGD1, Stargardt disease retinal organoids carrying the c.768G>T silent variant.

## Discussion

Non-coding alterations that disrupt pre-mRNA processing represent a major yet often underrecognized contributor to the hidden heritability of RGDs, as supported by literature indicating that splicing variants may account for pathogenesis in up to 30% of RGD cases.^3^ Notably, such splicing defects can potentially be rescued by steric-blocker ASOs targeting cryptic splice sites.

In this study, we applied an integrated genomic and functional approach to resolve the molecular diagnoses of two families with IRDs and to assess the therapeutic potential of ssASOs for correcting splicing defects in three different IRDs. GS allowed the detection of three cryptic variants in *SDCCAG8* and *CEP290*. A third family carrying the recurrent *ABCA4* c.768G>T silent variant^26^^,^^27^ was included to corroborate the translational strength of our pipeline and to provide an additional model for RNA-based therapeutic correction. By combining *in silico* prediction tools with experimental RNA analyses, we functionally validated the two novel *SDCCAG8* cryptic variants, c.740+267C>T and c.740+288G>C, both resulting in inclusion of the 112 bp pseudoexon ψ7a and generation of the aberrant isoforms 7-ψ7a-8 and 7-ψ7a-7b-8. Regarding the *CEP290* intronic variant c.6136−436A>G and the recurrent *ABCA4* silent variant c.768G>T, we reproduced the splicing anomalies previously reported,^25^^,^^26^^,^^27^ thereby validating the suitability of LCA fibroblasts and HeLa cells expressing the ABCA4 minigene as cellular models for ASO testing.

These findings strongly support the concept that cryptic splicing variants account for a substantial fraction of unresolved cases and highlight the necessity of GS-based diagnostic workflows capable of interrogating non-coding regions. Furthermore, consistent with growing evidence that deep intronic variants often remain undetected or unprioritized by ES,^28^ all pathogenic variants identified here were located far from canonical splice sites. Interestingly, one of the two novel *SDCCAG8* variants identified in this study, c.740+267C>T, was predicted to be benign by both HSF and SpliceAI, despite its clear involvement in pseudoexon inclusion demonstrated experimentally. This discrepancy underscores the current limitations of *in silico* tools in identifying non-canonical splicing defects, particularly when variants act through complex regulatory mechanisms or cooperate with additional sequence changes. Our findings reinforce the importance of integrating computational prediction with functional RNA or minigene assays, as reliance on bioinformatic prioritization alone may lead to the exclusion of clinically relevant pathogenic variants.

In the illustrative families, identifying the precise splicing alteration was not only diagnostically meaningful but also essential for designing and validating targeted therapeutic molecules. Detailed characterization of each pseudoexon, including the cryptic acceptor and donor splice sites involved, provided the basis for rational ssASO design. Importantly, the variant-specific nature of these splicing defects enabled the development of personalized therapeutic strategies tailored to each patient’s unique molecular lesion. Thus, achieving a molecular diagnosis is no longer merely the end of the diagnostic journey but rather the foundation upon which personalized therapeutic strategies can be developed. A central outcome of this study is the demonstration that splicing anomalies can be effectively corrected using ssASOs.

In the SLSN family, *SDCCAG8*-ssASOs AC3 and AC4 significantly increased the physiological transcript while reducing both aberrant ψ7a-containing isoforms in patient-derived fibroblasts in a clear dose-dependent manner. For *CEP290*, all seven designed ssASOs efficiently suppressed the ψ44-containing isoform and restored physiological splicing to near-control levels in LCA patient fibroblasts. Notably, the *CEP290*:c.6136−436A>G variant has been identified *in trans* with a pathogenic variant in two additional Italian LCA probands^25^ (S. Banfi, G. Piluso, M. Karali, unpublished data), further supporting the translational relevance of the proposed molecules. For *ABCA4*, all five ssASOs tested (DO2, DO3, DO6, DO7, and DO8) partially rescued the physiological b1-6-b2 isoform in the minigene system. The validation of the two most effective molecules (DO3 and DO8) in patient-derived ROs further supports the therapeutic potential of our molecules. Notably, our preliminary data indicate that the efficacy of ssASOs may be substantially enhanced by employing 2′MOE/PS chemistry. Of note, a recent study reporting successful ASO-mediated correction of the recurrent *ABCA4* c.768G>T variant^27^ further corroborates the feasibility and clinical promise of our splicing-modulation strategy. In both Karjosukarso et al.^27^ and the current study, therapeutic ASOs targeted the cryptic donor splice site located 34 (−2 of the donor motif) and 35 (−1 of the donor motif) nt downstream of the canonical site. Compared with the previously published molecule,^27^ our ASOs are designed to span the cryptic donor splice site, positioning it near the center of the oligonucleotide sequence (positions 15/16 in DO3 and 14/15 in DO8).

This study has some limitations. First, although patient-derived fibroblasts provided robust and scalable cellular models for the initial assessment of ssASO efficacy, future studies will be required to validate the most promising *SDCCAG8*- and *CEP290*-targeting molecules in ROs. Such models more closely recapitulate the cellular architecture and splicing environment of photoreceptors and retinal pigment epithelium, thereby enabling a more accurate evaluation of therapeutic efficacy in disease-relevant tissues. Furthermore, while effective ssASOs were identified for all three splicing defects investigated, the partial restoration of physiological splicing observed for *SDCCAG8* and *ABCA4* indicates that further optimization may be beneficial. Although the design and screening of additional ssASOs could potentially improve splicing correction, future efforts may benefit more from targeted strategies, such as fine sequence optimization of candidates (“microwalking”) around relevant splice-regulatory elements and the exploration of alternative chemistries.

Overall, our results underscore that splicing variants leading to the activation of cryptic acceptor and/or donor splice sites are highly actionable, as ssASOs can precisely mask pathological splice sites or regulatory elements with unique specificity.

The successful application of ssASOs across three distinct genes and pathogenic mechanisms highlights the broader therapeutic promise of RNA-targeting drugs. The speed and modularity of ssASO design, combined with the increasing availability of tissue-relevant models such as patient-derived fibroblasts and ROs, form the basis of a scalable and translational pipeline for monogenic diseases. Our data add to the growing clinical experience indicating that RNA-based therapies are ushering in a new era of genetically guided treatments, in which pathogenic mechanisms identified through GS directly inform the design of individualized therapeutics.

Although ssASOs offer strong translational potential, several challenges must still be addressed before their clinical application becomes widespread. Efficient delivery remains a major barrier for many tissues; however, the eye represents an advantageous target for RNA therapeutics, as local administration can achieve high retinal exposure while limiting systemic distribution. Because *SDCCAG8*, *CEP290*, and *ABCA4* are predominantly expressed in photoreceptors and their connecting cilium or outer segments, optimization of outer retinal drug delivery will be an important consideration for future clinical translation. Intravitreal injection currently represents the most clinically established approach for ASO delivery in IRDs, as demonstrated by the clinical development of sepofarsen for *CEP290*-associated LCA.^15^^,^^16^ Another consideration is the inherent need for patient- or variant-specific molecules, as each ssASO must precisely mask the aberrant splice site or pseudoexon responsible for the defect. This requirement increases both regulatory and manufacturing complexity, and the development of standardized discovery-to-therapy pipelines will be crucial for ensuring feasibility in *n*-of-few therapeutic settings. Finally, the reversibility of vision loss and the timing of intervention are critical biological determinants of therapeutic success. SLSN, LCA, and STGD1 differ in disease onset and progression, and the availability of intact photoreceptors at the time of treatment will heavily influence the extent of functional rescue achievable, as already seen from clinical experience in administering the voretigene neparvovec treatment in IRD patients with bi-allelic *RPE65* variants.^29^^,^^30^^,^^31^ Longitudinal natural history and *in vivo* studies will be needed to establish the therapeutic window and the degree of vision recovery possible across these disorders.

Overall, this study demonstrates that deep-intronic cryptic splicing variants represent a significant source of hidden heritability in inherited eye disorders and that the resulting splicing defects can be effectively targeted using ssASOs. By integrating GS-based variant discovery, functional splicing assays, and rational ASO design, we established an end-to-end translational workflow capable of moving from diagnosis to therapy development. These findings reinforce the feasibility of RNA therapeutics for RGDs and support the growing vision of a precision-medicine framework in which individualized therapies are derived directly from each patient’s genomic information.

## Materials and methods

### Samples and genetic studies

Within the PNNR-PARADIGM, a national initiative aimed at establishing an end-to-end pipeline—from molecular diagnosis to therapeutic intervention—for non-coding mutations in rare diseases, we investigated three patients from two independent families with a clinical diagnosis of IRD and carrying cryptic variants in known IRD genes, namely *SDCCAG8* (GeneBank: NM_006642.5; family SLSN) and *CEP290* (GeneBank: NM_025114.4; family LCA). In addition, a third IRD family carrying a previously described silent variant^26^^,^^27^ in the *ABCA4* (GeneBank: NM_000350.3; family STGD1) gene was also included in the study (Table 1). Dermal fibroblasts were obtained from SLSN and LCA probands by skin biopsy. For the STGD1 proband, hiPSCs were acquired from the Stem Cell Technology Center at Radboud University Medical Center in Nijmegen, the Netherlands.^32^ All analyses were performed after obtaining informed consent. The study conformed to the Declaration of Helsinki and was approved by the Ethics Committee of the IRCCS Mondino Foundation and Sant’Orsola Hospital (Comitato Etico di Area Vasta Emilia Centro N° 01/2023/Sper/AOUBo).

### *In silico* predictions

SpliceAI (https://spliceailookup.broadinstitute.org/) and HSF (https://hsf.genomnis.com/home) were used to predict the potential impact of the identified variants on splicing. Additionally, HSF and SpliceAid (https://www.introni.it/splicing.html) were exploited to evaluate splicing regulatory elements (SREs) within the variant-containing regions (Table 2).

### Cell culture

Fibroblasts and HeLa cells were maintained in Dulbecco’s modified Eagle’s medium (DMEM; Thermo Fisher Scientific) supplemented with 10% fetal bovine serum (FBS; Thermo Fisher Scientific), 1% penicillin-streptomycin (Thermo Fisher Scientific), and 1 mM sodium pyruvate (Thermo Fisher Scientific). Cells were cultured at 37°C and in the presence of 5% CO_2_.

### Characterization of splicing anomalies associated with cryptic variants

To evaluate splicing patterns associated with cryptic variants, two complementary strategies were used depending on RNA availability. For patients with SLSN and LCA, total RNA was extracted from fibroblasts using the Direct-Zol RNA MiniPrep system (Zymo Research), and cDNA was generated using the M-MLV Reverse Transcriptase kit (Invitrogen). Target regions were PCR amplified starting from 50 ng of cDNA and using 0.5 μM of custom primers (Table 3), KAPA2G Buffer A 5× (Sigma-Aldrich), 0.2 mM dNTPs (Thermo Fisher Scientific), and 5 U KAPA2G Fast HotStart DNA Polymerase (Sigma-Aldrich). Touchdown cycling was used, beginning with 14 cycles starting at an annealing temperature of 63°C for 15 s, followed by 25 cycles with an annealing temperature of 56°C for 15 s, and elongation at 72°C for 15 s each cycle. PCR products were visualized on a 1% or 1.5% agarose gel. To resolve and characterize the different splicing isoforms present in each PCR product, amplicons were subjected to TA cloning, and individual white colonies were screened using vector-based primers. In brief, PCR products were purified (DNA Clean&Concentrator kit; Zymo Research) and ligated into the pGEM-T Easy Vector System I (Promega) using T4 DNA Ligase (Promega). Plasmids were transformed using single-use JM109 competent cells (Promega). After blue/white screening with IPTG (Promega) and X-Gal (Promega), PCR was performed on white colonies using pGEM-specific primers SP6 and T7 (Table 3), followed by Sanger sequencing. For the STGD1 patient, fibroblasts or fresh blood samples were not available. Splicing characterization was therefore performed by minigene assays as previously described.^33^ Briefly, the genomic region containing the candidate variant and flanking intronic sequences was PCR amplified from patient and control DNA samples using the KAPA2G Fast HotStart DNA Polymerase kit (Sigma-Aldrich) and custom primers (Table 3). The PCR products were then cloned into a pGEM-T Easy Vector (Promega) and transformed into JM109 competent *E*. *coli* cells, following the manufacturer’s protocol. Plasmids containing either the WT (from the control; TA-ABCA4-WT) or the mutated sequence (from the proband; TA-ABCA4-MUT) were extracted using ZymoPURE Plasmid MiniPrep (Zymo Research) and subsequently subcloned into the pSPL3 vector (Life Sciences-Invitrogen). The final pSPL3 plasmids (+ABCA4-WT or -MUT) were transfected into HeLa cells using Lipofectamine 2000 (Thermo Fisher Scientific). After 36 h, total RNA was extracted using the Direct-Zol RNA MiniPrep system (Zymo Research), cDNA was synthesized using the M-MLV Reverse Transcriptase kit (Invitrogen) and PCR amplified with primers designed on the synthetic exons of the pSPL3 vector (SD6 and SA2; Table 3). The PCR products were purified using the DNA Clean & Concentrator kit (Zymo Research) and Sanger sequenced with Big Dye Terminator chemistry (Thermo Fisher Scientific) to characterize splicing outcomes.

**Table 3:** List of primers

ID	Primer sequence (5′-3′)
**Characterization of splicing anomalies associated with cryptic variants**	

RT-PCR experiments	
c.SDCCAG8-7F	TGAGGAAAAGTGTGAAATTGAGG
c.SDCCAG8-8R	CAAACAAAGACCACCAACACG
c.CEP290-43F	CAACTGGCATGACTGTTGATCAGG
c.CEP290-46R	TCTAACATGGCCAAGTTTCCGC
c.ABCA4-5F	CACTATTTCTCATTAAAAACATCGGCC
c.ABCA4-8R	GGAGGTCAGACAGGATGCCC
pGEM-T cloning	
SP6	TAATACGACTCACTATAGGG
T7	CATACGATTTAGGTGACACTATAG
gDNA_ABCA4_ex6-EcoR1-F	ggt**GAATTC**GTGGCTTTAATCCCATCTGC
gDNA_ABCA4_ex6R-BamH1-R	ggt**GGATCC**GGTTGAGACCATTTCCAGAGG
MINIGENE (pSPL3) cloning	
SD6-F	TCTGAGTCACCTGGACAACC
SA2_R	ATCTCAGTGGTATTTGTGAGC
SD6-R	TTGTGGGTTGGGGTCTGTGG

**3D retinal organoids generation and ASO treatment**	

Syber Green-based qPCR assays	
cABCA4-ex6F (assay 1)	TCTGTATGCCAACGTGGACT
cABCA4-ex7R (assay 1)	GGCTGTCTAGGAGTGTGGGA
cABCA4-ex5F (assay 2)	CTCTCAAGTCCGTCCAGAGC
cABCA4-ex6R (assay 2)	TGCAGGCGATGTCCTTCAG

Primers used for each experimental step are listed.

### ssASO design

Secondary structures of the target pre-mRNA regions surrounding each cryptic variant were analyzed using SFold (http://sfold.wadsworth.org/cgi-bin/index.pl) and RNAstructure v.6.5 (Mathews Lab 2021) (http://rna.urmc.rochester.edu/RNAstructure.html), considering partially opened and partially closed regions as target regions.^34^ A total of 21 ASOs for *SDCCAG8* variants, 11 for the *CEP290* variant, and 9 for *ABCA4* were designed and evaluated by secondary analyses (Table 4). Specifically, the CG content and melting temperature were calculated using the oligonucleotide properties calculator tool (http://biotools.nubic.northwestern.edu/OligoCalc.html). Free energy of the ASO, the ASO-ASO complexes, and the binding energy of the ASO and its target sequence were validated using RNAstructure, v.6.5 (Mathews Lab 2021). The off-target analysis was performed using Standard Nucleotide BLAST of NCBI. ASOs were synthesized by Kaneka Eurogentec S.A (Belgium; https://www.eurogentec.com/en/) with 2′OMePS or 2′MOE/PS modifications.

**Table 4:** List of splice-switching antisense oligonucleotides designed to target the cryptic splicing events identified in the three ED families

Gene	Strategy (target site)	ASO ID	ASO sequence	%GC	Free energy of the ASO	Free energy of the ASO-ASO complexes	Binding energy of the ASO and its target sequence	Off targets
**Therapeutic ssASOs**								

SDCCAG8	cryptic ASS of ψ7a	AC1	CUUAUUUCUACAAGAAAACAC	not met	/	/	/	/
SDCCAG8		AC2	GAUUCUUAUUUCUACAAGAAAAC	not met	/	/	/	/
SDCCAG8^a^		AC3^a^	CCCGGCCGUGAUUCUUAUUUCUAC^a^	met	met	met	met	Met
SDCCAG8^a^		AC4^a^	CCGGCCGUGAUUCUUAUUUCUACAAG^a^	met	met	met	met	Met
SDCCAG8	cryptic DSS of ψ7a	DO5-7a	GAAACGGGGUUUCACCAUGUUGGC	met	met	met	not met	Not met
SDCCAG8		DO6-7a	CGGGGUUUCACCAUGUUGGCCAGG	met	met	not met	/	Not met
SDCCAG8		DO7-7a	GUUUCACCAUGUUGGCCAGGAUGG	met	met	not met	/	Not met
SDCCAG8		DO8-7a	GGUUUCACCAUGUUGGCCAGGAUG	met	met	not met	/	Not met
SDCCAG8		DO9-7a	CACCAUGUUGGCCAGGAUGGUCUC	met	not met	/	/	/
SDCCAG8	c.740+267C>T	MUT1-10	CUGGGAUUACAGGCAUGAGCCACC	met	met	met	met	Not met
SDCCAG8		MUT1-11	GCUGGGAUUACAGGCAUGAGCCAC	met	not met	/	/	/
SDCCAG8		MUT1-12	GUGCUGGGAUUACAGGCAUGAGCC	met	not met	/	/	/
SDCCAG8	c.740+288G>C	MUT2-13	GCACUUUCGGAGGCUGAAGCGGG	not met	/	/	/	/
SDCCAG8		MUT2-14	CCAGCACUUUCGGAGGCUGAAGCG	not met	/	/	/	/
SDCCAG8		MUT2-15	CUGUAAUCCCAGCACUUUCGGAGG	met	met	met	not met	/
SDCCAG8		MUT2-16	CACUUUCGGAGGCUGAAGCGGGCG	not met	/	/	/	/
SDCCAG8		MUT2-17	GUAAUCCCAGCACUUUCGGAGGC	met	met	met	met	Not met
SDCCAG8	cryptic DSS of 7b	DO18-7b	GUACUCACCUUAUGCUUUAGUUUAC	not met	/	/	/	/
SDCCAG8^a^		DO19-7b^a^	GGGUACUCACCUUAUGCUUUAG^a^	met	met	met	met	Met
SDCCAG8^a^		DO20-7b^a^	CGUUUAUGGGUACUCACCUUAUGC^a^	met	met	met	met	Met
SDCCAG8		DO21-7b	CUCACCUUAUGCUUUAGUUUACGUC	met	met	met	not met	/
CEP290	cryptic ASS of ψ44	AC1	GACUGGUAUCCUUAUGAAAAGAG	met	met	met	met	Not met
CEP290^a^		AC2^a^	GUGACUGGUAUCCUUAUGAAAAG^a^	met	met	met	met	Met
CEP290^a^		AC3^a^	CGACUGUGACUGGUAUCCUUAUG^a^	met	met	met	met	Met
CEP290		AC4	CCUAUUCGACUGUGACUGGUAUCC	met	met	met	met	Met
CEP290^a^	cryptic DSS of ψ44	DO5^a^	CAUAAUUUCCAGUACCUCAGAAUG^a^	met	met	met	met	Met
CEP290^a^		DO6^a^	GUCAUAAUUUCCAGUACCUCAG^a^	met	met	met	met	Met
CEP290		DO7	GUACCUCAGAAUGUGACUUUAUUUG	not met	/	/	/	/
CEP290		DO8	CCAGUACCUCAGAAUGUGAC	met	met	met	met	Not met
CEP290^a^	c.6136−436A>G	MUT9^a^	GACUGGGUCUUCACAGAGGUAUUC^a^	met	met	met	met	Met
CEP290^a^		MUT10^a^	CUGGGUCUUCACAGAGGUAUUCAAG^a^	met	met	met	met	Met
CEP290^a^		MUT11^a^	GGAGACUGGGUCUUCACAGAGG^a^	met	met	met	met	Met
ABCA4	cryptic DSS of ir6	DO1	CCCAGGAAUCACCUUGCAAUUGGC	met	met	met	met	Not met
ABCA4^a^		DO2^a^	CACCUUGCAAUUGGCGAGCAGCC^a^	met	met	met	met	Met
ABCA4^a^		DO3^a^	GGAAUCACCUUGCAAUUGGCGAGC^a^	met	met	met	met	Met
ABCA4		DO4	CACCUUGCAAUUGGCGAGCAGC	met	not met	met	not met	Met
ABCA4		DO5	CUGCUACCCCAGGAAUCACCUUGC	met	met	met	not met	Not met
ABCA4^a^		DO6^a^	CCCCAGGAAUCACCUUGCAAUUGG^a^	met	met	met	met	Met
ABCA4^a^		DO7^a^	CCAGGAAUCACCUUGCAAUUGGCG^a^	met	met	met	met	Met
ABCA^a^		DO8^a^	CAGGAAUCACCUUGCAAUUGGCGAG^a^	met	met	met	met	Met
ABCA4		DO9	CACCUUGCAAUUGGCGAGCAGC	met	not met	met	not met	Met

**Scramble ASOs and positive control ASO for ABCA4**								

SCR 22	N/A	N/A	CAAUUGUAACCCGUUAAUGCCU	N/A	N/A	N/A	N/A	N/A
SCR 23	NA	N/A	GUUUAAUCUUCAUGAAAGGACGG	NA	N/A	NA	N/A	NA
SCR 24	N/A	N/A	GUGUCGUAGUGAGUGACUCCAAGC	N/A	N/A	N/A	N/A	N/A
SCR 25	N/A	N/A	GGACCGUGUCCUGAGCUCGAAUAAA	N/A	N/A	N/A	N/A	N/A
SCR 26	NA	N/A	GCUGUAGCCAUUCUAUGAUCGCUUCA	NA	N/A	NA	N/A	NA
ABCA4 C+	N/A	N/A	CCUUGCAAUUGGCGAGCAGCC (from Farnoodian et al.^23^)	N/A	N/A	N/A	N/A	N/A

Secondary analysis results for each ssASO are provided. Cutoff values were applied according to the criteria described by Aartsma-Rus.^34^ ASOs were synthesized with 2′-O-methyl RNA phosphorothioate (2′OMePS) modifications. ASS, acceptor splice site; DSS, donor splice site; ir, intron retention; /, analysis not performed; N/A, not applicable.

a Molecules selected for synthesis and subsequent experimental evaluation.

### *In vitro* validation of ssASOs

Fibroblasts and HeLa cells were seeded at a concentration of 3 × 10^4^ or 1.2 × 10^5^ cells/well, respectively, in 24-well plates and incubated 24 h before treatment. Fibroblasts at ∼80% confluence were left untreated (mock) or transfected with therapeutic or scramble ASOs at four different concentrations (20, 35, 50, and 65 nM) using Lipofectamine 2000 (Thermo Fisher Scientific).

HeLa cells were first transfected with 1,000 ng of pSPL3 plasmids containing the WT or the mutated *ABCA4* sequence and, after 24 h, with the therapeutic or scramble ASOs as described for fibroblasts. Cells were harvested 48 h post-transfection for RNA extraction, cDNA synthesis, PCR, and Sanger sequencing using gene-specific primers or pSPL3-specific primers (Table 3). For each ssASO and condition, at least three independent biological replicates were performed. Semi-quantitative analysis of amplicons was conducted using ImageLab (Bio-Rad). For each sample, all bands were quantified by evaluating their volume (intensity), with the total signal per lane considered as 100%; subsequently, the percentage contribution of each band within the lane was calculated.

### 3D RO generation and ASO treatment validation

Directed differentiation of patient-derived hiPSCs from the STGD1 proband (ST1, homozygous for the c.768G>T; Table 1) and a control cell line^32^ into 3D ROs was carried out as described in Brillante et al.^35^ 130-day-old ROs were left untreated (mock) or gymnotically treated twice per week for 4 weeks (8 total treatments) with 10 μM 2′OMePS ssASOs (equimolar pool of DO3 and DO8 ssASOs), a positive control (C+) ASO from Karjosukarso et al.,^27^ or scramble molecules (Table 4). The C+ molecule was also synthesized as a 2′MOE/PS ASO. Five days after the final treatment, RNA was extracted (miRNeasy Micro Kit, Qiagen), reverse transcribed, and PCR amplified using *ABCA4*-specific primers (Table 3), followed by Sanger sequencing. At least three independent biological replicates were analyzed per condition. Semi-quantitative analysis of amplicons was conducted using ImageLab (Bio-Rad), as previously described.

### Quantitative and semi-quantitative real-time PCR

The expression levels of *SDCCAG8* and *CEP290* and the reference gene *HMBS* were measured using predesigned TaqMan Gene Expression assays (Thermo Fisher Scientific; assay IDs: SDCCAG8 Hs00394863_m1, CEP290 Hs01565804_m1, and HMBS Hs00609297_m1). Relative expression levels were calculated using the comparative Ct (2^∧^−ΔΔCt) method.

Reactions were carried out in triplicate using the CFX Connect Real-Time system (Bio-Rad) and TaqMan Gene Expression Master Mix (Thermo Fisher Scientific), according to the manufacturer’s instructions.

Treatment-mediated modulation of splicing isoforms on *ABCA4* ROs was evaluated by SYBR Green-based qPCR assays using PowerUp SYBR Green Master system (Thermo Fisher Scientific). TA-ABCA4-WT and TA-ABCA4-MUT plasmids were used to generate a standard curve. Following quantification with a Qubit instrument (Life Technologies), plasmids were mixed at defined WT:MUT ratios (100:0, 90:10, 75:25, 50:50, 25:75, 10:90, and 0:100). Plasmids were analyzed (Figure S1) on a CFX Connect Real-Time PCR system (Bio-Rad) according to the manufacturer’s instructions. cDNA samples from ROs were tested by qPCR, and normalized d(RFU)/dT values were interpolated against the standard curve to determine WT:MUT transcript ratios.

### Statistical analysis

Statistical analyses and graphs were generated using GraphPad Prism v.8.4.2 (GraphPad software, San Diego, CA). Data are presented as mean ± SEM for biological replicates and standard deviation (SD) for technical replicates. Significance was assessed using a two-tailed Mann-Whitney *t* test. Values are expressed relative to scramble ASOs using the average of three independent experiments and at least three technical replicates.

## Data and code availability

The authors declare that all data generated or analyzed during this study and supporting the findings of the study are available within the paper and its supplemental information files.

## Acknowledgments

The Project is funded by the European Union’s NextGenerationEU – NRRP M6C2 Investment 2.1: Enhancement and strengthening of biomedical research in the NHS (Project code PNRR-MR1-2022-12376747, CUP E13C22003170007) E.G. acknowledges funding from #NEXTGENERATIONEU (10.13039/100031478NGEU) and the Italian Ministry of University and Research (MUR), National Recovery and Resilience Plan (NRRP), project MNESYS (PE0000006) – A Multiscale integrated approach to the study of the nervous system in health and disease (DN. 1553 11.10.2022), and from the Italian Ministry of Education, University, and Research (10.13039/501100003407MIUR), “Dipartimenti di Eccellenza (2023–2027) to the department of Molecular Medicine, University of Pavia.” M.A.D. and I.B. acknowledge funding from the European Union's Next Generation EU Mission 4 Component 2 Investment 1.3 (CUP D93C22000930002). We acknowledge Dr. Frans Cremers and Dr. Silvia Albert from the Radboud University Medical Center for providing human iPS cell lines.

## Author contributions

M. Zadorozhna performed the experiments and wrote the paper; A.B., G. Pezzin, and R.V. contributed to the cloning procedures; E.T., S.R., and I.T. generated the ROs and performed ssASO validations; M. Zadorozhna, I.B., and M.A.D. designed the ssASO molecules; F.S., F.I., E.L., E.P., M.K., M.S., and E.R. collected the samples; M. Zanobio, T.P., G.P., and S.B. performed NGS and analyzed the data; E.G. designed and supervised the research and wrote the paper, with input from all authors; M.A.D., S.B., G.P., I.T., and M.K. revised the manuscript; and E.G., T.P., I.T., and G.P. contributed to funding acquisition and project coordination.

## Declaration of interests

The authors declare no conflicts of interest.

## Supplemental information

Document S1. Figures S1 and S2 and Tables S1–S5

Document S2. Article plus supplemental information
