# Supplementary material for: Targeting cryptic splicing variants in SDCCAG8, CEP290, and ABCA4: A translational workflow from diagnosis to ASO-based therapy
Source: Mol Ther Nucleic Acids. 2026 Oct 14;37(4):103139. doi: 10.1016/j.omtn.2026.103139 (PMC13756157; doi:10.1016/j.omtn.2026.103139)
Supplement: Supplementary file 1 [file mmc1.pdf]

OMTN, Volume *37*

Supplemental information

Targeting cryptic splicing variants in *SDCCAG8*, *CEP290*, and *ABCA4*: A translational workflow from diagnosis to ASO-based therapy

Mariia Zadorozhna, Simona Rossi, Erika Tenderini, Ilaria Brentari, Anna Basile, Giulia Pezzin, Rebecca Venturini, Federica Isidori, Elena Luppi, Emanuele Panza, Fabio Sirchia, Elena Rossi, Mariateresa Zanobio, Marianthi Karali, Tommaso Pippucci, Giulio Piluso, Michela Alessandra Denti, Marco Seri, Sandro Banfi, Ivana Trapani, and Elisa Giorgio

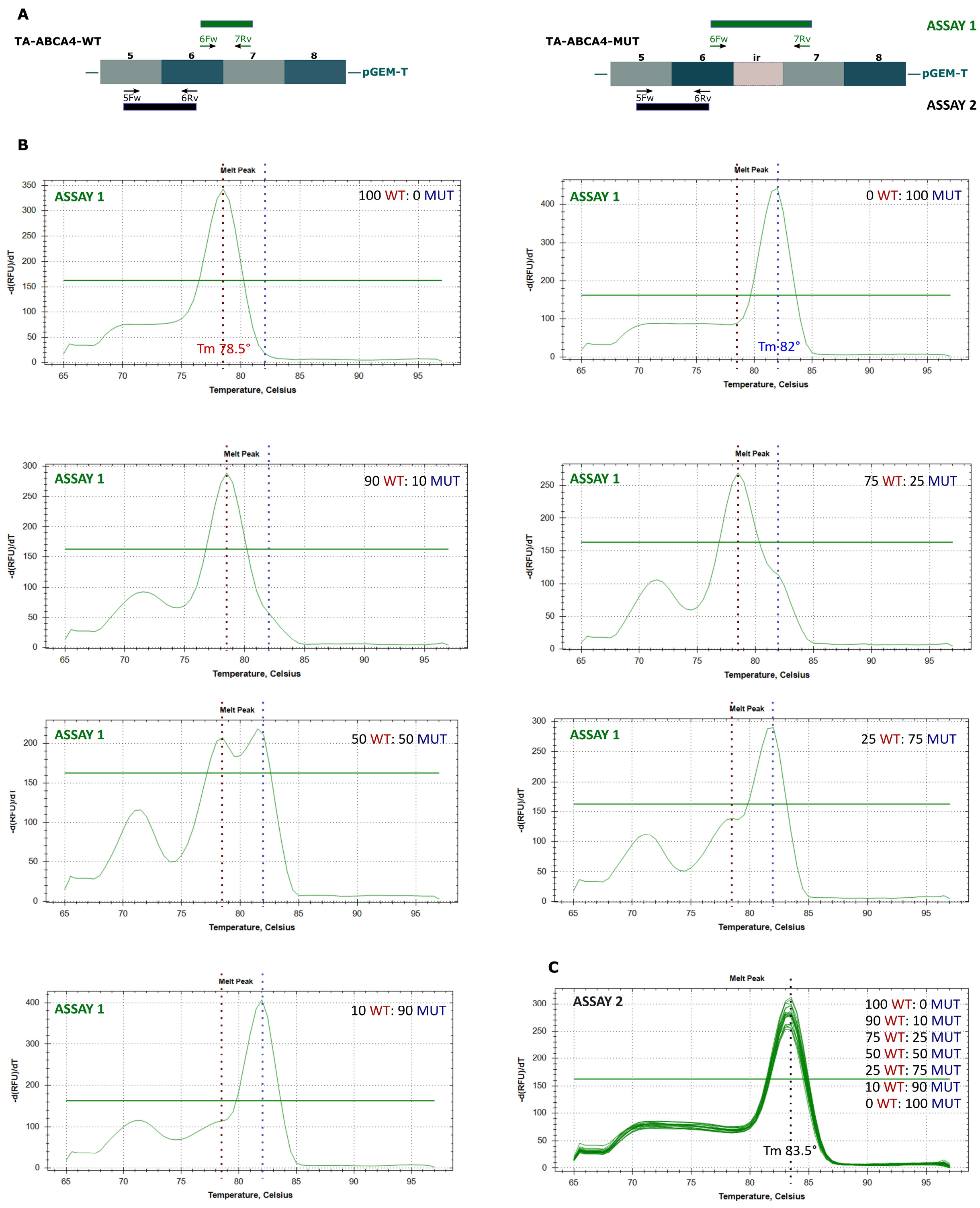

Figure S1. SYBR Green-based qPCR assay of ABCA4 splicing isoforms discrimination. (A) Schematic representation of SYBR Green assays designed. Assay 1 amplifies the exon 6–7 junction, enabling discrimination between WT and MUT isoforms, whereas Assay 2 targets the exon 5–6 junction. (B) TA-ABCA4-WT and TA-ABCA4-MUT plasmids were mixed at defined WT:MUT ratios to generate a standard curve. Representative melting peaks obtained for each tested ratio are shown. For Assay 1, the melting temperature (Tm) of the physiological isoform (ex6–7) is 78.5 °C (red dotted line), whereas the mutated isoform (ex6–i6–7) shows a Tm of 82 °C (light blue dotted line). Normalized d(RFU)/dT values at Tm 78.5 °C and 82 °C were used to generate the standard curve (see Fig. 4D). For Assay 2, the amplicon displays a Tm of 83.5 °C in all samples tested.

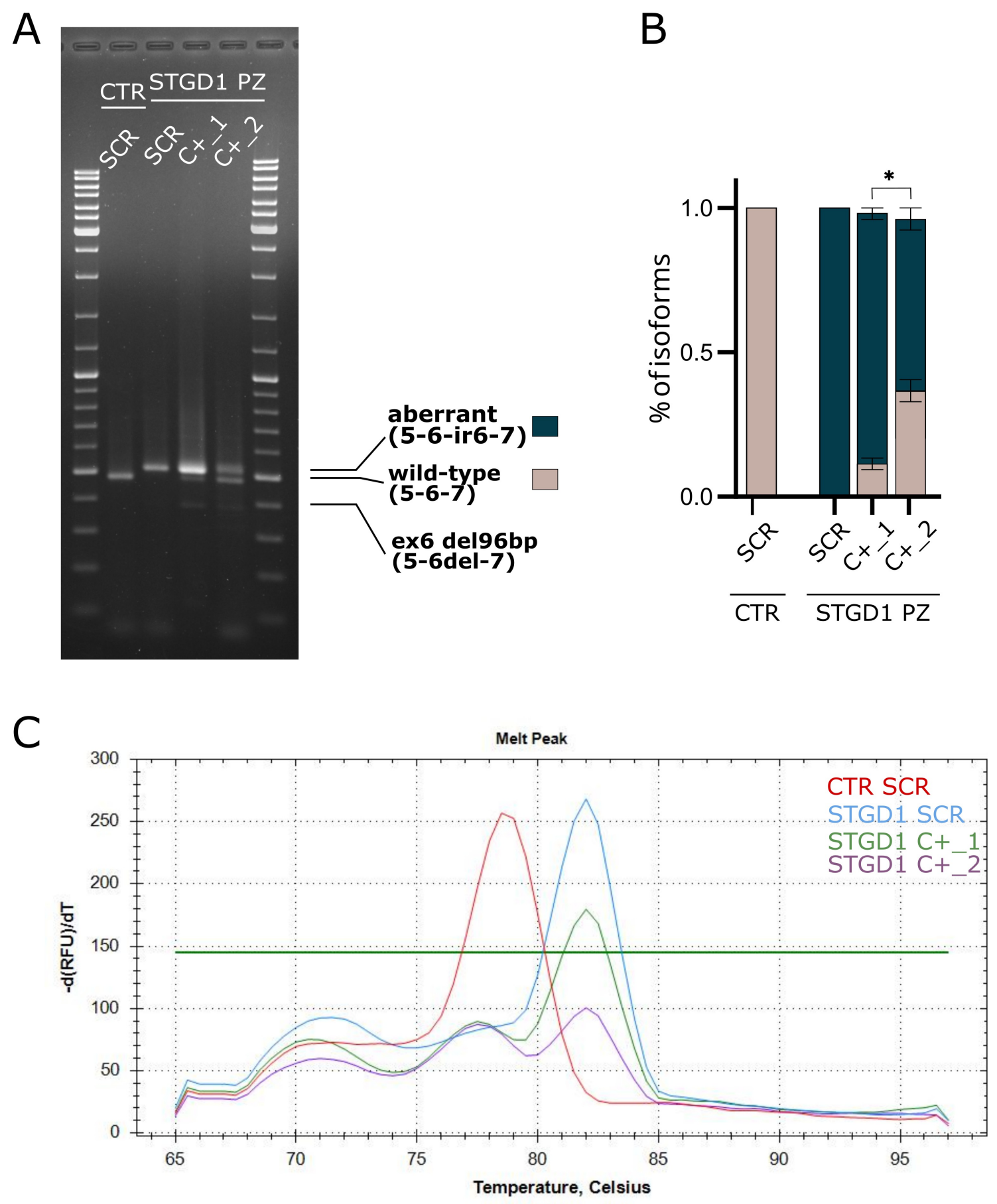

Figure S2. Effect of C+ ASO chemical modifications on ABCA4 splicing in hiPSC-derived retinal organoids. 130-day-old retinal organoids (ROs) derived from a STGD1 patient were gymnotically treated with scramble ASO (SCR) or positive control ASOs (C+_1 and C+_2, from [26]) at a final concentration of 10 µM, administered twice per week for four weeks. Electrophoretic analysis of RT-PCR products (**A**) and semi-quantitative densitometric analysis of splicing isoforms (**B**) are shown. Statistical significance (indicated by asterisks) was determined by comparing C+_2-treated samples with C+_1-treated samples (*P < 0.05; **P < 0.01; two-tailed Mann-Whitney test). (**C**) Representative SYBR Green–based qPCR melting curves for control (CTR) and STGD1 samples are shown. Melting peaks for the physiological isoform (WT) correspond to a Tm of 78.5°C (red dotted line), whereas the mutated isoform (MUT) corresponds to a Tm of 82°C (light blue dotted line). CTR: control retinal organoids; STGD1: Stargardt disease retinal organoids carrying the c.768G>T silent variant. C+_1: 2'-O-methyl RNA phosphorothioate C+ ASO; C+_2 2'-O-methoxyethyl phosphorothioate C+ ASO.

Table S1. Ratio of the physiological isoform in SLSN (SDCCAG8-mutated) fibroblasts before and after ssASO-treatments (48h).

	**CTRL**	**SLSN**	**ASO treated SLSN (20nM)**						
	mock	mock	SCR 22	SCR 24	SCR 26	AC3	AC4	DO19	DO20
**Mean**	0.895	0.323	0.348	0.337	0.320	0.839	0.849	0.483	0.416
**± SEM**	0.034	0.047	0.069	0.064	0.053	0.050	0.059	0.055	0.064
**p-value** (vs PZ scramble)	0.0079	0.8413				0.0079	0.0079	0.3095	0.3095
	**CTRL**	**SLSN**	**ASO treated SLSN (35nM)**						
	mock	mock	SCR 22	SCR 24	SCR 26	AC3	AC4	DO19	DO20
**Mean**	0.890	0.273	0.350	0.281	0.349	0.806	0.825	0.499	0.499
**± SEM**	0.026	0.071	0.061	0.027	0.035	0.054	0.046	0.066	0.070
**p-value** (vs PZ scramble)	0.0079	0.4206				0.0079	0.0079	0.3095	0.0317
	**CTRL**	**SLSN**	**ASO treated SLSN (50nM)**						
	mock	mock	SCR 22	SCR 24	SCR 26	AC3	AC4	DO19	DO20
**Mean**	0.913	0.375	0.384	0.331	0.331	0.800	0.788	0.588	0.619
**± SEM**	0.027	0.072	0.040	0.054	0.034	0.029	0.032	0.041	0.037
**p-value** (vs PZ scramble)	0.0079	> 0.9999				0.0079	0.0079	0.0079	0.0079
	**CTRL**	**SLSN**	**ASO treated SLSN (65nM)**						
	mock	mock	SCR 22	SCR 24	SCR 26	AC3	AC4	DO19	DO20
**Mean**	0.918	0.233	0.227	0.315	0.343	0.697	0.706	0.458	0.496
**± SEM**	0.028	0.054	0.020	0.044	0.029	0.041	0.031	0.035	0.031
**p-value** (vs PZ scramble)	0.0079	0.4206				0.0079	0.0079	0.0079	0.0317

Table S2. Ratio of the physiological isoform in LCA (CEP290-mutated) fibroblasts before and after ssASO-treatments (48h).

	**CTRL**	**LCA**	**ASO treated LCA (20nM)**										
	mock	mock	SCR 22	SCR 23	SCR 24	SCR 25	AC2	AC3	DO5	DO6	MUT9	MUT10	MUT11
**Mean**	1	0.658	0.773	0.671	0.723	0.731	0.987	0.997	0.997	0.989	0.991	0.992	0.985
**± SEM**	0	0.075	0.036	0.043	0.008	0.015	0.007	0.003	0.003	0.007	0.009	0.007	0.014
**p-value** (vs PZ scramble)	0.0079	0.3095					0.0079	0.0079	0.0079	0.0079	0.0079	0.0079	0.0079
	**CTRL**	**LCA**	**ASO treated LCA (35nM)**										
	mock	mock	SCR 22	SCR 23	SCR 24	SCR 25	AC2	AC3	DO5	DO6	MUT9	MUT10	MUT11
**Mean**	1	0.743	0.790	0.735	0.698	0.676	0.991	0.996	0.990	0.987	0.997	0.994	0.993
**± SEM**	0	0.048	0.029	0.050	0.020	0.046	0.003	0.002	0.004	0.008	0.001	0.002	0.004
**p-value** (vs PZ scramble)	0.0079	0.6905					0.0079	0.0079	0.0079	0.0079	0.0079	0.0079	0.0079
	**CTRL**	**LCA**	**ASO treated LCA (50nM)**										
	mock	mock	SCR 22	SCR 23	SCR 24	SCR 25	AC2	AC3	DO5	DO6	MUT9	MUT10	MUT11
**Mean**	1	0.642	0.648	0.632	0.676	0.689	0.988	0.992	0.990	0.992	0.998	0.969	0.992
**± SEM**	0	0.042	0.073	0.046	0.031	0.031	0.006	0.005	0.006	0.004	0.001	0.025	0.008
**p-value** (vs PZ scramble)	0.0079	>0.9999					0.0079	0.0079	0.0079	0.0079	0.0079	0.0079	0.0079
	**CTRL**	**LCA**	**ASO treated LCA (65nM)**										
	mock	mock	SCR 22	SCR 23	SCR 24	SCR 25	AC2	AC3	DO5	DO6	MUT9	MUT10	MUT11
**Mean**	1	0.701	0.624	0.547	0.624	0.568	0.984	0.969	0.990	0.995	0.997	0.997	0.995
**± SEM**	0	0.031	0.062	0.028	0.066	0.058	0.015	0.025	0.004	0.002	0.002	0.001	0.003
**p-value** (vs PZ scramble)	0.0079	0.3095					0.0079	0.0079	0.0079	0.0079	0.0079	0.0079	0.0079

Table S3. Ratio of the physiological isoform in Hela cells transfected with the pSPL3+ABCA4-WT or -MUT plasmid before and after ssASO-treatments (48h).

	**WT**		**MUT**		**ASO treated MUT (20nM)**							
	mock		mock		SCR 23	SCR 24	SCR 25	DO2	DO3	DO6	DO7	DO8
**Mean**	1		0		0	0	0	0.161	0.285	0.245	0.203	0.336
**± SEM**	0		0		0	0	0	0.022	0.029	0.020	0.014	0.023
**p-value** (vs PZ scramble)								0.0079	0.0079	0.0079	0.0079	0.0079
	**WT**		**MUT**		**ASO treated MUT (35nM)**							
	mock		mock		SCR 23	SCR 24	SCR 25	DO2	DO3	DO6	DO7	DO8
**Mean**	1		0		0	0	0	0.397	0.473	0.383	0.379	0.450
**± SEM**	0		0		0	0	0	0.119	0.122	0.053	0.057	0.113
**p-value** (vs PZ scramble)								0.0079	0.0079	0.0079	0.0079	0.0079
	**WT**		**MUT**		**ASO treated MUT (50nM)**							
	mock		mock		SCR 23	SCR 24	SCR 25	DO2	DO3	DO6	DO7	DO8
**Mean**	1		0		0	0	0	0.321	0.363	0.347	0.392	0.463
**± SEM**	0		0		0	0	0	0.099	0.088	0.056	0.077	0.086
**p-value** (vs PZ scramble)								0.0079	0.0079	0.0079	0.0079	0.0079
	**WT**		**MUT**		**ASO treated MUT (65nM)**							
	mock		mock		SCR 23	SCR 24	SCR 25	DO2	DO3	DO6	DO7	DO8
**Mean**	1		0		0	0	0	0.420	0.363	0.339	0.390	0.372
**± SEM**	0		0		0	0	0	0.125	0.110	0.114	0.110	0.119
**p-value** (vs PZ scramble)								0.0079	0.0079	0.0079	0.0079	0.0079

Table S4. SDCCAG8 and CEP290 mRNA expression in SLSN and LCA fibroblasts before and after ssASO-treatments (20nM; 48h)

SDCCAG8	**CTRL**		**SLSN**		**ASO treated SLSN**							
	mock		mock		SCR	AC3	AC4	DO19	DO20			
**Mean**	1.003		0.133		0.220	0.995	1.311	0.748	0.550			
**± SEM**	0.034		0.007		0.020	0.048	0.040	0.032	0.005			
**p-value** (vs PZ SCR)	0.0022		0.0022			0.0022	0.0022	0.0022	0.0022			
CEP290	**CTRL**		**LCA**		**ASO treated LCA**							
	mock		mock		SCR	AC2	AC3	DO5	DO6	MUT9	MUT10	MUT11
**Mean**	1.001		0.228		0.305	0.764	0.755	0.394	0.609	0.497	0.626	0.676
**± SEM**	0.022		0.043		0.008	0.057	0.111	0.012	0.049	0.031	0.039	0.051
**p-value** (vs PZ SCR)	0.0357		0.3929			0.0238	0.0238	0.0238	0.0238	0.0238	0.0238	0.0238

Table S5. Ratio of the physiological isoform in STGD1 130-day old Retinal Organoids (ABCA4-mutated) before and after ssASO-treatments (10uM; 4 weeks).

A) *Densitometric analyses*	**CTR**	**STGD1**	**ASO treated STGD1**			
	mock	mock	SCR	ABCA4-ASOs	C+_1	C+_2
**Mean**	1	0	0	0.142	0.117	0.369
**± SEM**	0	0	0	0.025	0.018	0.038
**p-value** (vs STGD1 scramble)				0.0061	0.0159	
**p-value** (vs STGD1 C+_1)						0.0159

B) *Syber Green-based qPCR analyses*	**CTR**	**STGD1**	**ASO treated STGD1**			
	mock	mock	SCR	ABCA4-ASOs	C+_1	C+_2
**Mean**	0.956	0.826	0.080	0.182	0.206	0.362
**± SEM**	0.011	0.005	0.018	0.004	0.028	0.004
**p-value** (vs STGD1 scramble)				0.0286	0.0571	0.0286
